# Prostate Cancer Gleason Score From Biopsy to Radical Surgery: Can Ultrasound Shear Wave Elastography and Multiparametric Magnetic Resonance Imaging Narrow the Gap?

**DOI:** 10.3389/fonc.2021.740724

**Published:** 2021-11-23

**Authors:** Cheng Wei, Yilong Zhang, Xinyu Zhang, Wael Ageeli, Magdalena Szewczyk-Bieda, Jonathan Serhan, Jennifer Wilson, Chunhui Li, Ghulam Nabi

**Affiliations:** ^1^ Division of Imaging Sciences and Technology, School of Medicine, University of Dundee, Dundee, United Kingdom; ^2^ School of Science and Engineering, University of Dundee, Dundee, United Kingdom; ^3^ Division of Population Health and Genomics, University of Dundee, Dundee, United Kingdom; ^4^ Diagnostic Radiology Department, College of Applied Medical Sciences, Jazan University, Jazan, Saudi Arabia; ^5^ Department of Clinical Radiology, Ninewells Hospital, Dundee, United Kingdom; ^6^ Department of Pathology, Ninewells Hospital, Dundee, United Kingdom

**Keywords:** prostate cancer, ultrasound shear wave elastography, multiparametric MRI, PIRADS, radical prostatectomy, prostate biopsy

## Abstract

**Objectives:**

To investigate the impact of ultrasound shear wave elastography (USWE) and multiparametric magnetic resonance imaging (mpMRI) in predicting a change in biopsy-assigned Gleason Score (GS) after radical surgery for localised prostate cancer (PCa).

**Method:**

A total of 212 men opting for laparoscopic radical prostatectomy (LRP) between September 2013 and June 2017 were recruited into this study. All the participants had 12-core transrectal ultrasound (TRUS) biopsies and imaging using USWE and mpMRI before radical surgery. The predictive accuracy for imaging modalities was assessed in relation to upgrading and downgrading of PCa GS between the biopsies and radical prostatectomy using Student’s t-test and multivariable logistic regression analyses. A decision analysis curve was constructed assessing the impact of nomogram on clinical situations using different thresholds of upgrading probabilities.

**Results:**

Most GS 6 diseases on biopsies were upgraded on radical surgery (37/42, 88.1%). Major downgrading was seen in GS 8 category of disease (14/35; 37.1%), whereas no alteration was observed in GS 7 on biopsies in most men (55/75; 73.3%). In univariate analysis, higher preoperative prostate-specific antigen (PSA) (p = 0.001), higher prostate-specific antigen density (PSAD) (p = 0.002), stiffer USWE lesions (p = 0.009), and higher prostate imaging–reporting and data system (PIRADS) (p = 0.002) on mpMRI were significant predictors of upgrading. In multivariate logistic regression analyses, only PSA (p = 0.016) and USWE-measured tissue stiffness (p = 0.029) showed statistical significance in predicting upgrading.

**Conclusions:**

Measurement of tissue stiffness using USWE in clinically localised PCa can predict upgrading of GS and has the potential to improve patient management options.

## Highlights

○ Ultrasound shear wave elastography can significantly predict upgrading of biopsy-assigned Gleason Score in prostate cancer following radical surgery.
○ Ultrasound shear wave elastography is an emerging technology based on measurement of tissue stiffness.
○ USWE-measured tissue stiffness can impact decision analysis based on different probabilities of Gleason Score upgrading from biopsies to radical surgery in prostate cancer.

## Introduction

The histological Gleason Score (GS) obtained using 12-core transrectal ultrasound (TRUS) biopsy informs risk stratification and counselling of clinically localised prostate cancer (PCa) patients regarding various treatment options. The approach has significant limitations, as a large discrepancy exists between biopsy and postoperative radical prostatectomy GS in approximately 40% of all localised PCa patients especially for those with biopsy GS 6 disease (1–3). GS upgrading after radical surgery is also associated with poor disease prognostic factors such as extracapsular extension (ECE) and higher rates of biochemical recurrences (4). Thus, predicting GS prior to treatment of PCa becomes crucial, and the role of imaging as marker is less understood. The role of imaging in the detection and characterisation of PCa is now well-established (4). Prebiopsy multiparametric magnetic resonance imaging (mpMRI) has been widely applied to increase biopsy accuracy, particularly over the last decade (5–8). However, there is still a burgeoning interest in investigating the role that imaging can play in predicting underestimated GS in biopsies. This will help in accurately assessing prognosis, treatment selection, and decision-making.

Recently, ultrasound shear wave elastography (USWE) has emerged as a promising imaging modality in the detection and characterisation of localised PCa (9–11). USWE can assess tissue stiffness of the whole prostate including cancerous tissue. USWE measures the shear wave speed generated by specialised ultrasound transducers through the target organs. Under imaging, the speed of these scattered shear waves is shown as a colour-coded dynamic map of tissue stiffness (presented as Young’s modulus) in real time (12, 13). The USWE-based imaging approach not only provides characterisation of clinically significant PCa (9) but also predicts biochemical recurrence on follow-up (14).

Although previous studies have focused on other multifactorial analyses and nomograms to predict GS change after radical surgery (3, 15–21), USWE or mpMRI, key imaging modalities have seldom been considered as potential imaging markers to predict GS upgrading or downgrading in PCa (22).

The aim of this study was to assess the impact of imaging markers [tissue stiffness using USWE and prostate imaging–reporting and data system (PIRADS) using mpMRI] in predicting a change in biopsy-assigned GS after radical surgery for localised prostate cancer. Furthermore, we aimed to quantify the additional benefits that imaging information may bring to the already known and reported clinicopathological parameters through the construction of nomogram and decision-analysis curves.

## Materials and Methods

### Study Cohort

**Figure 1:**
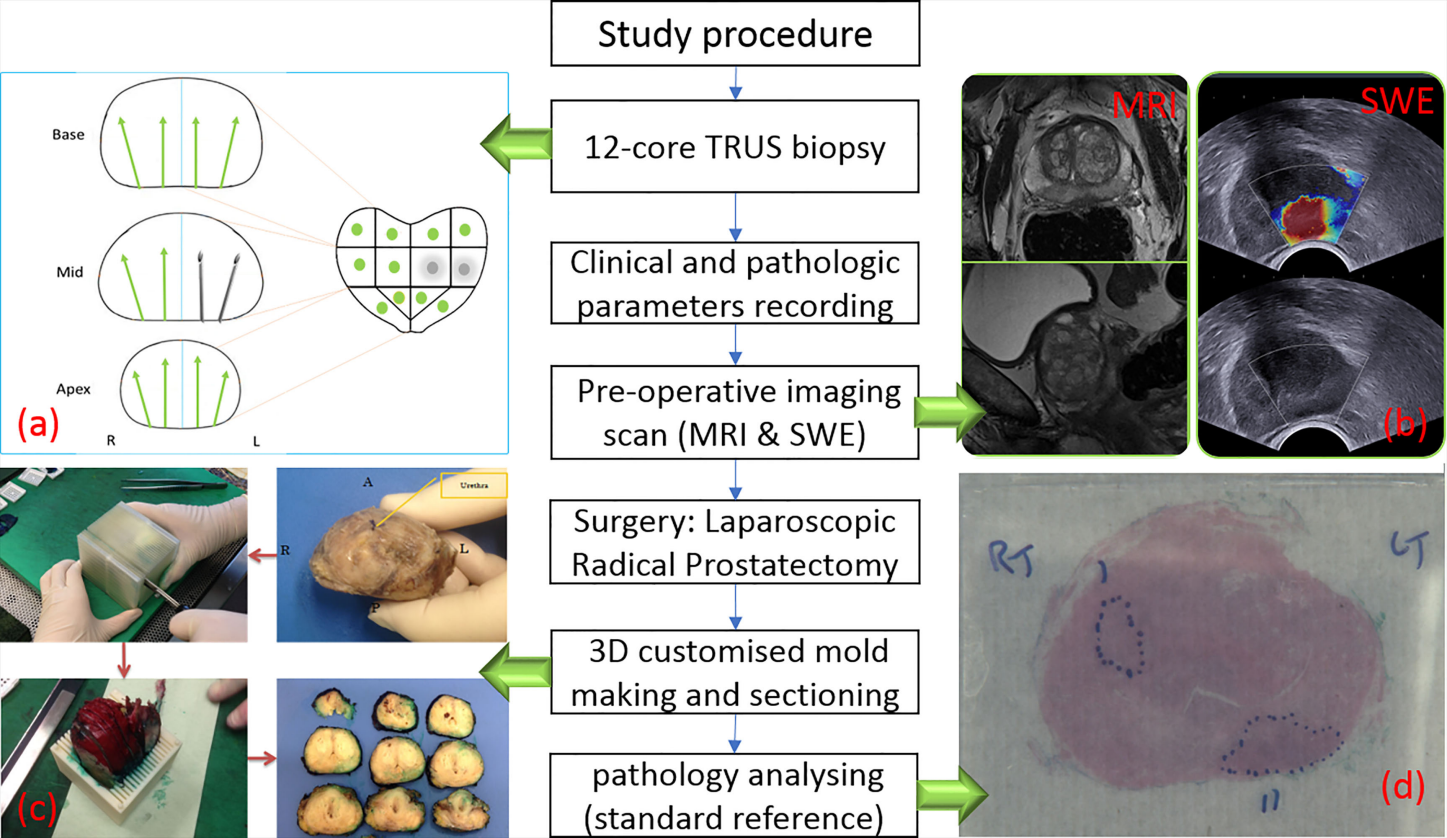
Flow chart of study procedure. **(A)** TRUS biopsy result with two positive biopsy cores. **(B)** One suspicious lesion in peripheral zone is shown in MRI (left) and SWE (right) images. **(C)** Post-prostatectomy specimen sectioning in steps (23). **(D)** Histopathology photo after analysing.

Two hundred and twelve patients opting for laparoscopic radical prostatectomy between September 2013 and June 2017 were recruited into this study. All patients were confirmed to have PCa on 12-core TRUS biopsies. In brief, transrectal ultrasound imaging of prostates was performed. After measuring size of prostate gland, local anaesthetic agent was infiltrated from the base to apex. Prostate was divided into 12 regions (as per our protocol) including lateral and paramedian regions. Each region was biopsied and sent for histopathology. Participants were then scanned using two imaging modalities: mpMRI and USWE preoperatively. The images from mpMRI were assessed, and abnormal areas were classified using PIRADS score by two uro-radiologists. The USWE images were analysed, and a quantitative cancer stiffness estimation in kilopascals (kPa) was made. Patients’ age at the time of radical surgery, prostate-specific antigen (PSA), prostate weight, prostate-specific antigen density (PSAD), biopsy GS, number of positive cores, maximum of cancer in cores, clinical stage, and postoperatively pathological GS were collected and analysed. Prostate specimens were sectioned in a 3D printed patient specific mould and analysed by two experienced study pathologists including the co-author (JW with more than 5 years’ experience) (23, 24). **Figure 1** shows the flow of participants recruited to the study. **Table 1** presents baseline patient characteristics.

**Table 1:** Patient characteristics.

Age (years)	
Median (IQR)	67.0 (63.8–72.0)
Mean (SD)	67.2 (5.7)
Range	44.0-77.0
Prostate-specific antigen (ng/ml)	
Median (IQR)	9.4 (7.1–12.5)
Mean (SD)	11.4 (7.6)
Range	0.1–47.7
Clinical stage (%)	
≤T2a	148 (69.8%)
T2b/c	44 (20.8%)
T3	20 (9.4%)
Biopsy Gleason Score (%)	
≤6	42 (19.8%)
7 (3 + 4)	75 (35.4%)
7 (4 + 3)	39 (18.4%)
>7	56 (26.4%)
No. of positive cores	
Median (IQR)	4.0 (2.0–7.0)
Mean (SD)	4.8 (3.3)
Range	1.0–14.0
Maximum percentage of cancer pre-core (%)	
Median (IQR)	50.0 (20.0–80.0)
Mean (SD)	50.4 (30.0)
Range	5.0–100.0
The interval from biopsy to SWE (days)	
Median (IQR)	102.5 (83–118)
Mean (SD)	102.6 (27.3)
Range	46–189
The interval from biopsy to MRI (days)	
Median (IQR)	43 (35–48)
Mean (SD)	43.8 (11.2)
Range	21–78
Radical prostatectomy weight (g)	
Median (IQR)	59.5 (47.5–76.5)
Mean (SD)	66.9 (29.4)
Range	31.0–207.0

### USWE Protocol and Acquisition

All USWE images were obtained using a transrectal endocavitory ultrasound transducer (SuperSonic Imagine, Aix en Provence, France) with patients being in either lithotomy or lateral position the day before the scheduled radical surgery. USWE mode was activated, and prostate gland elastograms were obtained from the cranial to caudal direction for each lobe of the prostate. All regions were scanned as described in our previously published protocol (11). Guidelines for clinical practice have been framed based on data emanating from many centres (25). Each patient’s prostate gland was scanned transrectally; USWE images were acquired in transverse planes from the base to apex with a gap of 4–6 mm. The most suspicious cancer located in the planes was marked and reconstructed offline into 3D images. Suspicious areas for cancer were scanned by rotating the transducer in different directions to confirm abnormalities and to perform measurements of their sizes. Three stiffness measurements of shear wave speed in m/s or Young’s modulus in kPa using pseudo-colour maps were obtained independently by three researchers. The ratio between abnormal and normal areas were also recorded (**Figure 1B**).

### MRI Protocol and PIRAD Score

MRI scan of each patient was performed using 3T scanners (TIM Trio, Siemens, Erlangen, Germany) 6–8 weeks after the prostate biopsy procedure (26). The MRI protocol was derived from the European Society of Uro-radiology (ESUR) guidelines 2012 (27) for PCa detection; PIRADS v2.0 was applied in this study, and only PIRAD ≥3 lesions on MRI were marked and PIRADS 1 and 2 were taken as negative findings. All MR images were analysed and scored by two experienced uro-radiologists (MS-B and JS); both the radiologists were blinded to patients’ clinicopathology data.

### Statistical Analyses

A two-stage logistic regression process was used to investigate the explanatory factors that could predict upgrading of GS 6 or 7(3 + 4) and downgrading of GS 7(4 + 3) and above on biopsy. First, univariate logistic regression was applied to examine associations between single explanatory factor and the outcomes, respectively. Absolute percentages of each variable, univariate odds ratio (OR), 95% CI of univariate OR, and p-value were presented. Multivariate logistic regression was then applied to assess and adjust for significant predictive factors regarding patient characteristics. The predictive factors in the multivariate logistic regression model were a combination of significantly associated factors from the bi-variate logistic regression. Age, PSA, PSAD, maximum percentage of cancer in the core, and prostate gland weight, and quantitatively assessed stiffness using USWE were treated as continuous variables; clinical stage, number of positive cores for cancer, PIRADS scores, and GS were treated as ordinal variables. The reference groups of those predictive factors were set if they were considered as a meaningful reference of that variable. Adjusted OR, 95% CI of adjusted OR, and p-value were derived after multivariate logistic regression.

In addition, logistic regression model coefficients were used to derive a nomogram predicating the probability of GS upgrading or downgrading from biopsy. Non-informative or non-significant variables in univariate logistic regression for GS upgrading were removed. The bias-corrected calibrated values were generated from internal validation based on 200 bootstrap resamples. A decision-analysis curve was constructed assessing the impact of the nomogram using different threshold probabilities of upgrading or downgrading of GS. All analyses were performed using SPSS 22 (IBM Corporation, New York, USA) and R software (v 3.5.3). The alpha level was set at 0.05 to determine two-tailed significance.

## Results

### Change in GS in the Cohort From Biopsy to Radical Surgery

**Figure 2:**
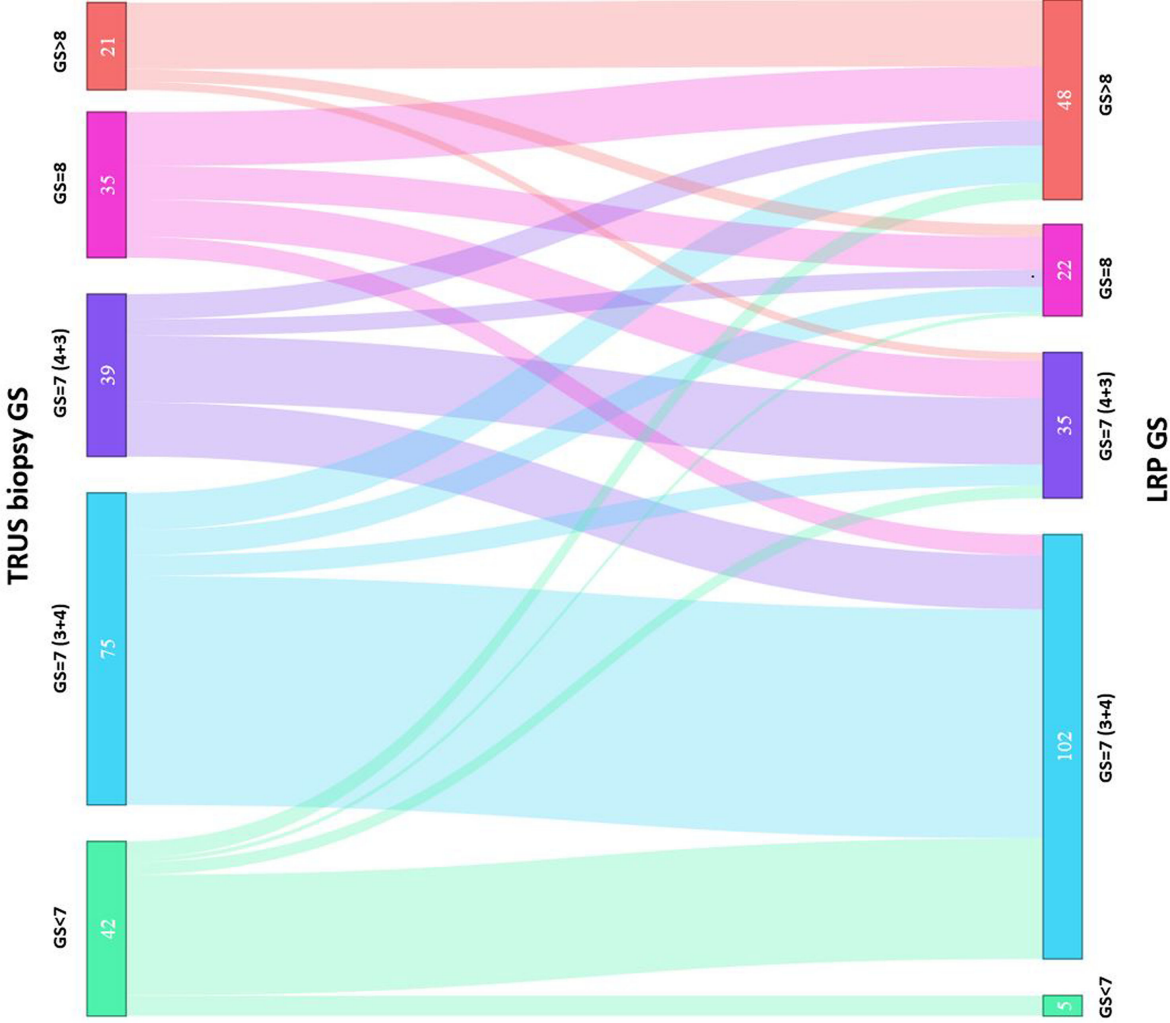
Sankey diagram of comparison between biopsy Gleason Score and prostatectomy Gleason Score.

A detailed map of biopsy GS and radical prostatectomy specimen GS is shown in **Table 2**. A Sankey diagram in **Figure 2** presents same data in an alternate way. No change in Gleason Score was seen in 47.2% of all the cases (100/212). Out of the 42 cases with GS 6 disease on biopsy, the majority (37/42, 88.1%) was upgraded following radical surgery. GS 3 + 4 disease on biopsies remained stable in most of the cases (55/75; 73.3%) and so did the GS 9 (4 + 5 or 5 + 4) disease in most cases (76.2%). GS 4 + 3 disease on biopsies had downgrading in one-third of cases (13/39, 33.3%). Most downgrading (13/35; 37.1%) was seen in biopsy GS 8 (3 + 5, 4 + 4, 5 + 3) category disease.

**Table 2:** Radical prostatectomy grades stratified by biopsy Gleason Scores.

LRP GS		Biopsy GS					Total
		<7	7 (3 + 4)	7 (4 + 3)	8	9–10	
**6**	**Count**	**5**	**0**	**0**	**0**	**0**	**5**
	% within LRP GS	100%	0.0%	0.0%	0.0%	0.0%	100%
	% within Biopsy GS	11.9%	0.0%	0.0%	0.0%	0.0%	2.4%
	% of total	2.4%	0.0%	0.0%	0.0%	0.0%	2.4%
**7 (3 + 4)**	**Count**	**29**	**55**	**13**	**5**	**0**	**102**
	% within LRP GS	28.4%	53.9%	12.7%	4.9%	0.0%	100%
	% within biopsy GS	69.0%	73.3%	33.3%	14.3%	0.0%	48.1%
	% of total	13.7%	25.9%	6.1%	2.4%	0.0%	48.1%
**7 (4 + 3)**	**Count**	**3**	**5**	**16**	**9**	**2**	**35**
	% within LRP GS	8.6%	14.3%	45.7%	25.7%	5.7%	100%
	% within biopsy GS	7.1%	6.7%	41.0%	25.7%	5.7%	16.5%
	% of total	1.4%	2.4%	7.5%	4.2%	0.9%	16.5%
**8**	**Count**	**1**	**6**	**4**	**8**	**3**	**22**
	% within LRP GS	4.5%	27.3%	18.2%	36.4%	13.6%	100%
	% within biopsy GS	2.4%	8.0%	10.3%	22.9%	14.3%	10.4%
	% of total	0.5%	2.8%	1.9%	3.8%	1.4%	10.4%
**9–10**	**Count**	**4**	**9**	**6**	**13**	**16**	**48**
	% within LRP GS	8.3%	18.8%	12.5%	27.1%	33.3%	100%
	% within biopsy GS	9.5%	12.0%	15.4%	37.1%	76.2%	22.6%
	% of total	1.9%	4.2%	2.8%	6.1%	7.5%	22.6%
**Total**	**Count**	**42**	**75**	**39**	**35**	**21**	**212**
	% within LRP GS	19.8%	35.4%	18.4%	16.5%	9.9%	100%
	% within biopsy GS	100%	100%	100%	100%	100%	100%
	% of total	19.8%	35.4%	18.4%	16.5%	9.9%	100%

LRP GS: laparoscopic radical prostatectomy Gleason Score; Biopsy GS: biopsy Gleason Score.

### Multifactorial Analysis of GS Change at Radical Surgery

**Table 3** shows the preoperative clinical and imaging parameters in men with and without upgraded GS at radical surgery. As seen, the data indicate that upgraded patients had a higher PSA level (p = 0.001) and a greater PSAD (p = 0.002), stiffer cancerous tissue as estimated by USWE (p = 0.009), and higher PIRADS 4/5 score (p = 0.002). The results also showed a trend that upgraded patients were older (p = 0.130), with more positive cores (p = 0.608), maximum percentage of cancer in a given core (0.071), and smaller prostates (p = 0.806), but none of these variables were statistically significant. In multivariate logistic regression analyses (**Table 4**), higher stiffness values at USWE (p = 0.029) and higher PSA level (p = 0.016) predicted upgrading from biopsy GS ≤ 7 (3 + 4) to GS ≥ 7 (4 + 3) after radical surgery. The PIRADS score at mpMRI failed to maintain the same significance (p < 0.05) in both univariate analysis (p = 0.056) and multivariate analysis (p = 0.068).

**Table 3:** Association of clinical and pathologic parameters with Gleason Score (GS) group: upgrading from biopsy GS ≤7(3 + 4) to GS ≥7(4 + 3) at radical prostatectomy.

variables	Upgrade (n = 28)	No-upgrade (n = 89)	t value (95%CI)	p-value
**Age, year**				
Median (IQR)	70.0 (65.0–72.0)	67.0 (63.0–71.0)	1.49 (−0.61, 4.14)	0.130
Mean (SD)	68.8 (5.5)	67.0 (5.3)		
**PSA, ng/ml**				
Median (IQR)	11.5 (7.3–16.2)	8.8 (6.9–10.5)	3.34 (1.98, 7.78)	0.001
Mean (SD)	14.5 (10.1)	9.6 (5.4)		
**No. of positive cores**				
Median (IQR)	3.0 (1.0–5.0)	3.0 (2.0–5.0)	0.47 (−1.07, 1.72)	0.641
Mean (SD)	4.0 (3.3)	3.6 (2.8)		
**Maximum % cancer/core**				
Median (IQR)	45.0(20.0–76.3)	30.0 (20.0–50.0)	1.82 (−0.98, 23.02)	0.071
Mean (SD)	48.2(32.0)	37.2 (26.6)		
**Pathology weight (continuous), gram**				
Median (IQR)	62.0 (51.0–76.5)	63.0 (47.6–86.8)	1.24 (−5.43, 23.73)	0.216
Mean (SD)	64.7 (18.3)	73.9 (36.8)		
**PSAD (continuous), ng/ml^2^**				
Median (IQR)	0.2 (0.1–0.2)	0.1 (0.1–0.2)	3.21 (0.04, 0.16)	0.002
Mean (SD)	0.3 (0.2)	0.2 (0.10)		
**Clinical stage (%)**				
≤T2a	16	69	1.83 (−0.02, 0.51)	0.070
T2b/c	9	14		
T3	3	6		
**USWE (continuous), kPa**				
Median (IQR)	145.1 (128.8–168.5)	128.7 (115.3–147.6)	2.64 (4.98, 34.84)	0.009
Mean (SD)	154.2 (42.3)	134.3 (31.4)		
**PI-RADS**				
≤3	1	19	2.23 (0.02, 0.34)	0.028
4 and 5	27	69		
Not reported	0	1		

**Table 4:** Univariate and multivariate logistic regression models to predict upgrading from biopsy GS ≤7 (3 + 4) to GS ≥7 (4 + 3) at radical prostatectomy.

	Univariate		Multivariate	
	OR (95% CI)	p-value	OR (95% CI)	p-value
Weight	0.991 (0.976–1.006)	0.218	–	–
SWE	1.015 (1.003–1.027)	0.014	1.015 (1.002–1.028)	0.029
PSA level (ng/ml)	1.098 (1.026–1.169)	0.007	1.087 (1.016–1.163)	0.016
PI-RADS				
≤3	1 (referent)	–	1 (referent)	–
>3	7.435 (0.948–58.305)	0.056	7.317 (0.862–62.097)	0.068
Positive Core	1.038 (0.900–1.198)	0.605	–	–
Percentage	1.014 (0.999–1.029)	0.074	–	–
Clinical stage				
T3	1 (referent)	–	–	–
T2b/c	2.156 (0.487–9.556)	0.312	–	–
≤T2a	0.778 (0.154–3.927)	0.761	–	–

OR: odd ratio; Univariate and Multivariate analysis are two statistical analyses. Univariate involves the analysis of a single variable while multivariate analysis examines two or more variables. Most multivariate analysis involves a dependent variable and multiple independent variables.

### Nomogram Construction, Validation, and Defining Thresholds for Decision Analysis

**Figure 3:**
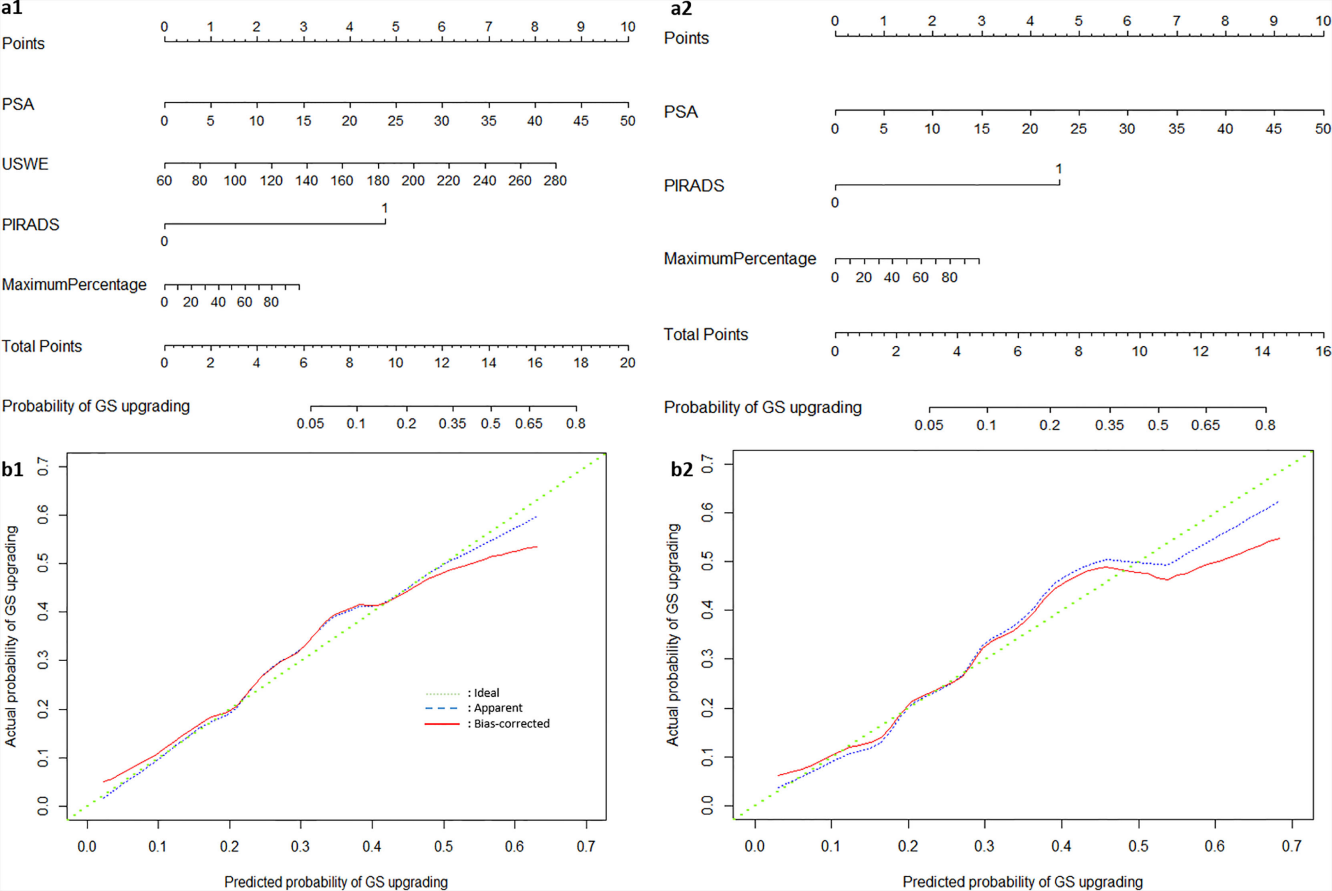
The nomograms of Gleason Score upgrading prediction with **(A1)** and without USWE score **(A2)**. Calibration plots of observed and predicted probability of GS upgrading with **(B1)** and without USWE score **(B2)**.

**Figure 4:**
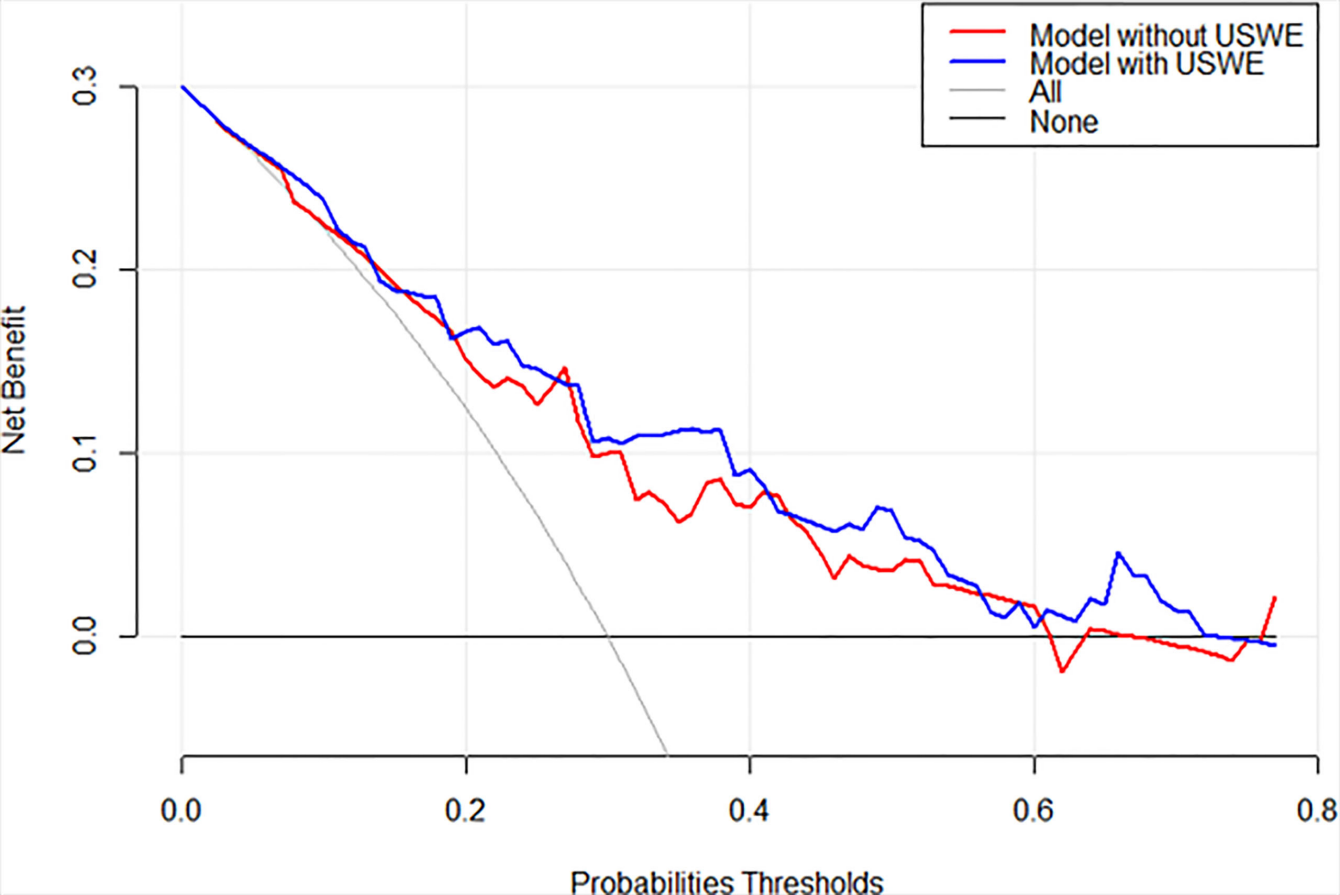
Decision analysis demonstrated a high net benefit of USWE score model across a wide range of threshold probabilities. Prediction model without USWE score (red line); prediction model with USWE score (blue line).

**Figures 3A1, A2** show a constructed nomogram predicting the upgrading of GS from biopsies to radical surgery with or without USWE data. Longer scales indicate a higher percentage of impact, and larger points suggest probability of upgrading. PSA level had the greatest impact in both nomograms. USWE counted as the second highest impact factor for GS upgrading. The nomograms were then internally validated using 200 bootstrap samples, and internal calibration curves were highlighted (**Figures 3B1, B2**). The calibration curves based on internal validation results are set for the probability of prediction at different levels. As seen, the curves demonstrated excellent agreement between the prediction according to the nomogram and actual observation. Decision analysis assumed that the threshold probability of a change in GS at which the clinician or patient would make an informed decision weighing the relative harms of a false-positive and a false-negative prediction using USWE information. A range of threshold probabilities was shown at which the magnitude of benefits of USWE was compared with no USWE information (**Figure 4**). The net benefit for the model using USWE was slightly higher but not quantitively proved at various thresholds compared with the model without USWE (blue *vs.* red line). The mean size of lesions from USWE was 16.1 ± 7.2 mm (range from 7.4 to 44.8 mm).

## Discussion

This was the first study to assess the role of both USWE and mpMRI in predicting change in biopsy-assigned GS following radical surgery in men presenting with clinically localised PCa. A review of the literature showed only limited reports of mpMRI parameters used in predicting GS upgrading. Lai et al. (28) found that mpMRI findings could predict upgrading GS 3 + 3 disease on first biopsies in men on active surveillance. Abd-Alazeez et al. (29) concluded that a patient with higher PIRADS score on mpMRI predicted a high likelihood of high GS disease at radical surgery in men with low-risk PCa in biopsy. No 3D fabricated moulds were used to orient imaging to histopathology in any of the reported studies, a clear contrast to the present study. Similar observations were made by our group in the past (22). In contrast and interestingly, Klotz et al. (30) observed in a randomised multicentre prospective trial that adding MRI to clinicopathological factors did not boost the prediction ability of biopsy-assigned GS. In our study, we observed a high number of patients with PIRADS 4 and 5 in upgraded than not-upgraded patients [28.1% (27/96) *vs.* 5.0% (1/20), p = 0.002], but it was not a significant predictor in either univariate logistic regression model (p = 0.056) or multivariate logistic model (p = 0.068). There are no reports in the literature of USWE imaging being used in predicting change in GS. Previous studies only reported USWE as a promising diagnostic modality in the detection of clinically significant PCa (9–11, 31, 32).

Preoperative PSA levels or PSAD are the most frequently analysed factors as predictor of GS change in the reported literature and were included in this study as well. From the reported publications (19, 21, 28, 33), it appears that PSA or PSAD performed consistently well, although in other studies, the significance was not as strong in comparison to other predictors (34, 35), but all the studies had used preoperative PSA in multivariate logistic regression models. In this study, PSA level was found to be one of the two significant parameters in multivariate logistic regression analysis.

Smaller prostate size was not a statistically significant predicting factor for GS upgrading in this study (p = 0.086). This is similar to observations by other studies (16, 18–20), although Freedland et al. (36) showed that decreased prostate size was associated with higher Gleason grade, more aggressive behaviour, and higher biochemical recurrence rates.

In studies by Epstein et al. (19) and Gondo et al. (33), age, PSA level, prostate weight, and maximum cancer core involvement were all statistically significant predictors of downgrading. In our study, downgraded patients were more likely to have a lower PSA level (8.1 *vs.* 10.6 ng/ml), but this was not statistically significant (p = 0.075). Three studies summarised downgrading from biopsy GS 3 + 4 to biopsy GS <7 at varying rates of 7.3%, 9.0%, and 12.0% (19, 33, 37), respectively. No patients’ postoperative GS was downgraded to GS 3 + 3 in our study (see first raw of **Table 2**).

There were limitations worth mentioning in this study. First, this study recruited men with histologically confirmed PCa and only those opting for radical surgery. The focus of the study was to obtain a robust reference standard of histology from radical prostatectomy obtained specimens. We used both USWE and MRI imaging modalities in a preoperative setting. This was considered as the standard of care approach at the time of study; however, this had the potential of introducing a selection bias in the study. Second, the biopsy technique used in this study was 12-cores TRUS biopsy without targeting, and this has potentially created sampling error in the patients recruited to this study. Third, MRI scans were obtained after biopsies confirmed PCa, and this might have introduced a detection bias in estimating PIRADS score. Finally, this was a single institutional study, and the findings require external validation and the reproducibility of the USWE technique (38). The use of USWE is not the standard of care, although guidelines and evidence are emerging in this area (39). The study was single centred with only operator performed USWE. Further reproducibility in multi-operator setting needs to be tested. We did not calculate the learning curve for this technology. Future studies could focus on the role of both USWE and/or MRI-targeted biopsy in patients suspected of PCa and in predicting change in GS from biopsies to radical surgery.

Measurement of tissue stiffness using USWE in clinically localised PCa can predict upgrading of GS and better guide patient management options. This information may help in counselling patients opting for PCa therapy for localised disease.

## Data Availability Statement

The datasets generated for this study are available on request to the corresponding author.

## Ethics Statement

The studies involving human participants were reviewed and approved by East of Scotland Research Ethics Service (EoSRES) REC 1. The patients/participants provided their written informed consent to participate in this study.

## Author Contributions

GN and CW contributed to the conception and design of the study. CW and YZ organised the database. YZ and XZ performed the statistical analysis. CW wrote the first draft of the manuscript. CW, WA, MS-B, JS, JW, and CL corrected and rewrote sections of the manuscript. All authors contributed to the article and approved the submitted version.

## Conflict of Interest

The authors declare that the research was conducted in the absence of any commercial or financial relationships that could be construed as a potential conflict of interest.

## Publisher’s Note

All claims expressed in this article are solely those of the authors and do not necessarily represent those of their affiliated organizations, or those of the publisher, the editors and the reviewers. Any product that may be evaluated in this article, or claim that may be made by its manufacturer, is not guaranteed or endorsed by the publisher.

## Abbreviations

GS, Gleason Score; TRUS, transrectal ultrasound; PCa, prostate cancer; mpMRI, multiparametric magnetic resonance imaging; USWE, ultrasound shear wave elastography; kPa, kilopascals; PSA, prostate-specific antigen; PSAD, prostate-specific antigen density; ESUR, European Society of Uro-radiology; PIRADS, prostate imaging–reporting and data system.
